# Supplementary material for: Ubiquitin turnover and endocytic trafficking in yeast are regulated by Ser57 phosphorylation of ubiquitin
Source: eLife. 2017 Nov 13;6:e29176. doi: 10.7554/eLife.29176 (PMC5706963; doi:10.7554/eLife.29176)
Supplement: Figure 1—source data 1. — Phosphorylation events that are elevated threefold or greater in Δppz1Δppz2 cells are shown. Also shown are phosphopeptides detected from Ppz1, which are present in wild-type cells and missing from Δppz1Δppz2 cells. [file elife-29176-fig1-data1.docx]

| **Table 1: SILAC analysis of *ppz* mutant phosphoproteome** | | |
| --- | --- | --- |
| Protein | **Function** | **Phosphosite (fold change in *ppz* mutant)** |
| Ppz1 | protein phosphatase | Ser250 (-12), Ser34 (-21) |
| Ato2 | ammonia transporter | Thr46 (35) |
| Bck2 | unknown | Ser334 (53) |
| Csf1 | unknown | Ser331 (8) |
| Ecm3 | unknown | Ser390 (3) |
| Ecm25 | Unknown | Ser555 (8) |
| Ecm30 | unknown | Ser1115 (3) |
| Emg1 | Methyltransferase | Thr179 (500) |
| Gde1 | GroPCho phosphodiesterase | Ser637 (500) |
| Hbt1 | unknown | Ser41 (4), Ser43 (4), Ser363 (4),  Ser671 (7), Ser1034 (7) |
| Hnm1 | choline transporter | Ser146 (21) |
| Jip4 | unknown | Ser360 (4), Thr662 (13) |
| Mdr1 | Rab GAP | Ser283 (54) |
| Miy1 | unknown | Ser343 (3) |
| Mlp1 | myosin-like protein | Thr337 (8) |
| Nup170 | Nuclear transport | Ser800 (500) |
| Pdc5 | pyruvate decarboxylase | Ser223 (36) |
| Pdr10 | ABC transporter | Thr852 (5) |
| Pob3 | FACT complex | Ser194 (500) |
| Pos5 | Mitochondrial NADH kinase | Thr222 (35) |
| Rex3 | RNA exonuclease | Ser65 (21) |
| Set3 | histone deacetylase | Ser236 (3) |
| Snd1 | protein secretion | Ser23 (8) |
| Spc110 | Spindle pole body component | Ser529 (8) |
| Trs130 | Golgi traffic | Ser259, Ser262 (12) |
| Tom20 | Mitochondrial import | Thr92 (54) |
| ubiquitin | degradation | Ser57 (3) |
| YGR035C | unknown | Ser55 (4) |
| YJR098C | unknown | Ser336 (500) |

**Figure 1, source data 1.** Results form SILAC-based quantitative comparison of the yeast phosphoproteome from wildtype (heavy) and *Δppz1Δppz2* (light) cells. Phosphorylation events that are elevated 3-fold or greater in *Δppz1Δppz2* cells are shown. Also shown are phosphopeptides detected from Ppz1, which are present in wildtype cells and missing from *Δppz1Δppz2* cells.
